# Contribution of *Plasmodium knowlesi* to Multispecies Human Malaria Infections in North Sumatera, Indonesia

**DOI:** 10.1093/infdis/jix091

**Published:** 2017-02-13

**Authors:** Inke N. D. Lubis, Hendri Wijaya, Munar Lubis, Chairuddin P. Lubis, Paul C. S. Divis, Khalid B. Beshir, Colin J. Sutherland

**Affiliations:** 1 Departments of Immunology and Infection and; 2 Pathogen Molecular Biology, London School of Hygiene & Tropical Medicine, London, United Kingdom;; 3 Department of Paediatrics, Faculty of Medicine, University of Sumatera Utara, Medan, Indonesia;; 4 Malaria Research Centre, Faculty of Medicine and Health Sciences, Universiti Malaysia Sarawak, Kota Samarahan, Malaysia

**Keywords:** Malaria, Indonesia, *Plasmodium knowlesi*.

## Abstract

**Background.:**

As Indonesia works toward the goal of malaria elimination, information is lacking on malaria epidemiology from some western provinces. As a basis for studies of antimalarial efficacy, we set out to survey parasite carriage in 3 communities in North Sumatera Province.

**Methods.:**

A combination of active and passive detection of infection was carried out among communities in Batubara, Langkat, and South Nias regencies. Finger-prick blood samples from consenting individuals of all ages provided blood films for microscopic examination and blood spots on filter paper. *Plasmodium* species were identified using nested polymerase chain reaction (PCR) of ribosomal RNA genes and a novel assay that amplifies a conserved sequence specific for the *sicavar* gene family of *Plasmodium knowlesi*.

**Results.:**

Of 3731 participants, 614 (16.5%) were positive for malaria parasites by microscopy. PCR detected parasite DNA in samples from 1169 individuals (31.3%). In total, 377 participants (11.8%) harbored *P. knowlesi*. Also present were *Plasmodium vivax* (14.3%), *Plasmodium falciparum* (10.5%) and *Plasmodium malariae* (3.4%).

**Conclusions.:**

Amplification of *sicavar* is a specific and sensitive test for the presence of *P. knowlesi* DNA in humans. Subpatent and asymptomatic multispecies parasitemia is relatively common in North Sumatera, so PCR-based surveillance is required to support control and elimination activities.

## Summary

Indonesia is aiming for malaria elimination by 2030. A parasitological survey of 3731 individuals in North Sumatera province used polymerase chain reaction to identify 1169 with parasitemia and highly specific amplification of *sicavar* genes to identify *Plasmodium knowlesi* infection in 377.

Malaria remains widespread across Southeast Asia. In Indonesia, 2 million cases of malaria are reported each year, with *Plasmodium falciparum* and *Plasmodium vivax* the 2 major reported causes [1]. Among other species contributing to human infections, *Plasmodium malariae* malaria may require hospitalization in the eastern province of Papua [2] but is not frequently encountered in western Indonesia. *Plasmodium knowlesi,* a parasite of long-tailed and pig-tailed macaques, is also known to infect humans. The morphological features in the blood stage are similar to those seen in *P. falciparum* and *P. malariae*, which in routine practice has led to frequent misdiagnosis [3–5]. High *P. knowlesi* parasitemia occurs in some individuals and has been reported to cause fatal disease [6]. Despite this, a proportion of *P. knowlesi* infections are asymptomatic and submicroscopic across all age groups [7]. A small number of human cases of *P. knowlesi* malaria have been documented in the province of Kalimantan, Indonesian Borneo [8] and in Aceh province [5], but this species has not yet emerged as a major cause of human malaria and is not considered in Indonesian government guidelines.

The Ministry of Health of Indonesia has implemented malaria control, aiming for elimination by 2030. Malaria surveillance relies on passive case detection by microscopic examination and rapid diagnostic tests (RDTs) at primary health care centers [9]. These tests are sufficient to detect clinical malaria infection caused by the 2 major species in Indonesia, *P. falciparum* and *P. vivax* [10]. However, identification of less common species, particularly at low-density parasitemia, is more difficult, which can lead to underdiagnosis [11]. Modeling of data from low-endemicity areas predicts that submicroscopic parasites may contribute 70%–80% of all malaria infections [12], and in vivo studies demonstrate that these contribute to ongoing malaria transmission [13]. Hence, the use of routine microscopy and RDTs in malaria surveillance fails to detect a substantial proportion of the human reservoir of infection and so may compromise malaria elimination strategies. One solution is to deploy molecular assays for parasite detection, because these can provide excellent sensitivity and specificity [14–17].

In preparation for a study of antimalarial drug efficacy in vivo, we performed intensive malaria screening in 3 regencies of the Province of North Sumatera, western Indonesia. In addition to microscopy, we used established polymerase chain reaction (PCR) assays [18]. However, these tests have limitations for the identification of *P. knowlesi* infection, because the target region of the 18S ribosomal RNA (rRNA) can cross-react with *P. vivax* [19]. Therefore, we developed a sensitive and highly *P. knowlesi*–specific nested PCR assay to ensure reliable determination of all *Plasmodium* species, including submicroscopic infection, in our study areas.

## METHODS AND MATERIALS

### Study Sites

**Figure 1.**
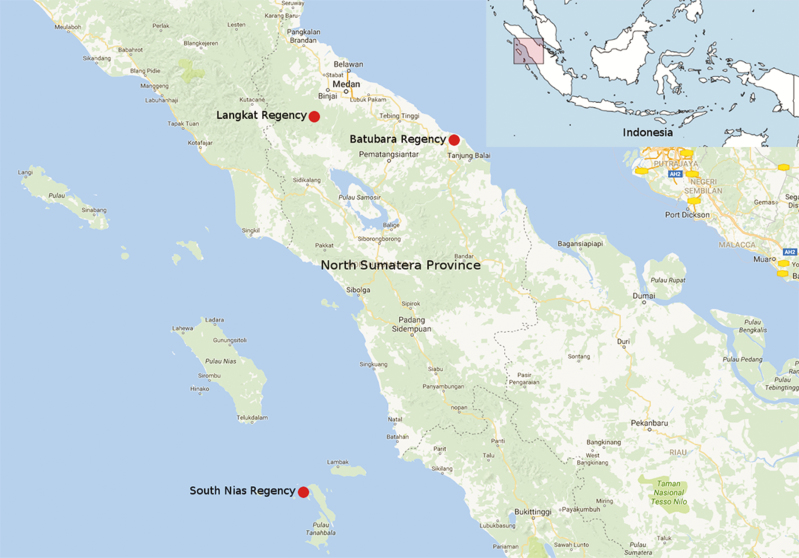
Map of North Sumatera province, Indonesia. The 3 studied regencies (Batubara, Langkat, and South Nias) are indicated.

A parasitological survey was conducted between January and June 2015 among persons attending outpatient clinics temporarily established in >80 localities across 3 selected regencies in North Sumatera province, Indonesia: Batubara, Langkat, and South Nias regencies (Figure 1). North Sumatera has a total area of 71 680.68 km^2^ with a population of 13 215 401. The province is subject to stable low malaria transmission and is currently planning for elimination by 2020. The 3 regencies were selected based on published malaria endemicity data [20]. Batubara, situated on the east coast facing peninsular Malaysia, comprises semiforested and plantation areas. Langkat is a forested highland area (altitude, 105–530 m above sea level), and South Nias is a cluster of islands in the Indian Ocean. Each regency is served by a district hospital and peripheral health clinics, but some rural villages in the study areas have very limited access to these services.

### Ethics Approval

The study was approved by the ethics committees of the University of Sumatera Utara, Indonesia (identifier 401/KOMET/FK USU/2014) and the London School of Hygiene & Tropical Medicine, United Kingdom (identifier 8504‑01).

### Sampling

Sampling strategies differed among the 3 sites, owing to contrasting geography and inconsistencies in access to health facilities. In Batubara, most communities had good access to a health clinic, which was open to patients from 8 am to noon, 6 days per week. We established a 24-hour, 7-day clinic for an 8-week period of screening, after intensive health promotion and education on malaria and sensitization concerning to our study objectives. This sensitization was facilitated by local leaders and carried out at the level of the whole community, but clinic attendance was entirely voluntary. Thus, sampling was not designed to reach the whole community. Local health clinics were also asked to refer patients with a malaria diagnosis to our team. Langkat is a forested area, with isolated villages that have poor access to health facilities; we therefore adopted a village-by-village approach, in which a sensitization meeting was followed by 2–3 days of screening, before the team moved to another village. The 31 communities we sampled in South Nias were spread across several islands, and sea journeys were required to move our team and the samples between each village and our study clinic/diagnostic laboratory, which was temporarily set up in a central location on Tello Island.

Malaria testing was done in 3 groups of persons. First, patients with fever (axillary temperature, ≥37.5°C) or a history of fever in the preceding 48 hours who presented at the health clinics were tested for malaria. Tests were also offered to healthy individuals who, after our community sensitization activity, came for a voluntary malaria check. This second group includes children who volunteered during school sensitization visits (Batubara and South Nias only). Third, household members of any individuals who were slide positive for malaria parasites were subsequently visited and also offered malaria testing. Finger-prick blood samples were taken from all participants for thick and thin blood films for microscopy examination (single reading), and blood was also spotted onto filter papers (3MM Whatman) for molecular analysis. Participants confirmed as malaria positive by microscopy were clinically assessed and treated according to national guidelines, regardless of symptoms.

### Laboratory Procedures

#### DNA Extraction

Parasite DNA was extracted from filter papers using the Chelex method [21, 22].

#### Parasite Species Identification by rRNA Gene Amplification

A conventional nested PCR assay targeting the genes encoding the *Plasmodium* 18S rRNA was performed on all samples for species determination and detection of submicroscopic infections [3, 18]. Positive controls for *P. falciparum*, *P. vivax*, *P. malariae*, *Plasmodium ovale* spp. and *P. knowlesi* were included in all nested PCR assays.

#### *Development and Validation of a Novel Highly Specific* P. knowlesi *PCR Assay*

To overcome the cross-reactivity between *P. knowlesi* and *P. vivax* that occurs in the rRNA gene PCR assays, we developed a heminested PCR assay based on a conserved region of the amino-terminal exon of the 50–70 members of the gene family encoding *P. knowlesi*–specific schizont-infected cell agglutination variant antigens (SICAvar) [23]. The following primers were used: first amplification, SICAf1, 5’-GGTCCTCTTGGTAAAGGAGG-3’and SICAr1, 5’- CCCTTTTTGACATTCGTCC-3’; second amplification, SICAf2, 5’-CTTGGTAAAGGAGGACCACG-3’ and SICAr1; these generated a final amplicon of 228–249 base pairs, encoding 76–83 amino acids. This sequence occurs in both types I and type II *sicavar* genes [23, supplementary table 1]. For the first round of amplification, 5 µL of DNA template was used in a total volume of 25 µL, and 0.2 µL of this product was the template for the 25-µL reaction mixture of the heminested amplification round. Amplifications were performed under the following conditions: 94°C for 3 minutes, then 30 cycles of 94°C for 30 seconds, 55°C for 30 seconds, and 65°C for 1 minute, with extension at 65 °C for 5 minutes.

This assay was tested against control DNA from all human malaria parasites (*P. falciparum*, *P. vivax*, *P. malariae*, *P. ovale curtisi,* and *P. ovale wallikeri*), simian malaria parasites (*P. knowlesi* and *Plasmodium inui*, *cynomolgi*, *coatneyi,* and *fieldi*), clinical *P. knowlesi* isolates obtained from Kapit of Malaysian Borneo, and human DNA from malaria-free individuals to assess specificity. To determine the limit of detection of the assay, *P. knowlesi* culture of known parasitemia (kindly provided by F. Mohring) was serially diluted in whole human blood and spotted on filter paper, and DNA was extracted with a QIASymphony automated DNA extraction system. All field isolates were tested for *P. knowlesi* infection using this novel PCR assay. The *sicavar* amplicons from a subset of samples were verified by direct sequencing using BigDye Terminator v3.1 cycle sequencing kits and analysis on an ABI 3730 Sequencer (Applied Biosystems). Results were aligned and compared with the *P. knowlesi* strain H reference genome, using Geneious (version 8.0.5) and BLAST (Basic Local Alignment Search Tool) software.

## RESULTS

### Validation of SICAvar Assay for *P. knowlesi–*Specific Identification

**Figure 2.**
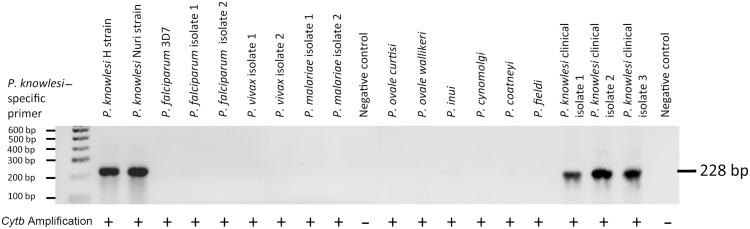
Validation of *Plasmodium knowlesi* primers targeting *sicavar* against human and simian malaria parasites reported from Southeast Asia. Control DNA of human malaria *Plasmodium* species were from imported cases in the United Kingdom (courtesy of the Public Health England Malaria Reference Laboratory); 2 isolates each are shown for *Plasmodium falciparum, Plasmodium vivax,* and *Plasmodium malariae*. Genus-specific primers for the cytochrome B gene (*cytb*) were used to confirm presence of detectable *Plasmodium* DNA in each sample, indicated by plus signs; bp, base pairs.

The novel SICAvar gene assay was validated against DNA from a range of *Plasmodium* species DNA and from in vitro cultured *P. knowlesi*. The test was found to be specific, because the primers did not generate bands from the DNA of any of the *Plasmodium* species tested other than *P. knowlesi* (Figure 2). The *sicavar*-targeted primers detected 377 *P. knowlesi* infections, suggesting significantly higher sensitivity than the rRNA assay, which identified only 76 *P. knowlesi*–infected individuals (Table 1). Furthermore, comparison of the results showed that only 10 persons (2.3%) had a positive test result in both assays. The finding that 66 individuals were *P. knowlesi* positive by rRNA nested PCR alone suggests that the test cross-reacts with *P. vivax* DNA in the absence of *P. knowlesi* under the conditions used in this study.

**Table 1.** Comparison of 2 PCR Assays for *Plasmodium knowlesi* Case Detection

Infection Detected	Cases, No. (%)			
	18S rRNA Assay	SICAvar Assay	Any Assay	Both Assays
Total *P. knowlesi* cases	76 (100)	377 (100)	443 (100)	10 (100)
*P. knowlesi* monoinfection	42 (55.3)	215 (57.0)	254 (57.34)	3 (30)
*P. knowlesi* plus *P. vivax*	16 (21.1)	65 (17.2)	77 (17.38)	4 (40)
*P. knowlesi* plus other *Plasmodium* spp. infections	18 (23.7)	97 (25.7)	112 (25.28)	3 (30)

Abbreviations: PCR, polymerase chain reaction; rRNA, ribosomal RNA; SICAvar, schizont-infected cell agglutination variant antigen.

Because we did not deploy a “tie-breaker” test for *P. vivax*, we cannot say for certain whether the *P. vivax* rRNA gene primers also cross-react with *P. knowlesi* DNA. We did find that 18 individuals were positive for both *P. vivax* and *P. knowlesi* rRNA amplicons in the nested PCR. Given this ambiguity, and the fact we were not able to repeat test the sample set, we proceeded with analysis taking the *sicavar* assay result as definitive for *P. knowlesi*. Nearly half of all *sicavar*-positive *P. knowlesi* infections were also rRNA amplicon positive for ≥1 other species, *P. vivax* being the most common coinfection (Table 1). The limit of detection of the assay, as performed on dried filter paper blood samples, was estimated as 0.1 parasite per µL of whole blood (data not shown).

**Figure 3.**
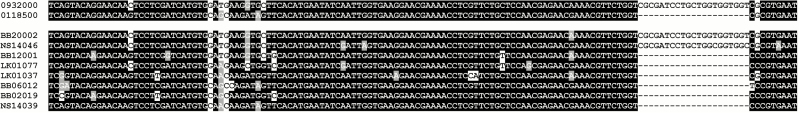
Sequence alignment of representative 120 and 141 nucleotide sequences of *sicavar* amplicons from the peripheral blood DNA of 4 participants from Batubara (BB) and 2 each from Langkat (LK) and South Nias (NS). Amplicons were produced by heminested polymerase chain reaction, as described in Materials and Methods. Sample order is determined by the alignment. Representative amplification products were chosen for this sample and sequenced directly using amplification primers to prime forward and reverse sequencing reactions. Sequences shown were confirmed in both directions. Two loci from the *Plasmodium knowlesi* strain H reference genome (PKNH_0932000, type I schizont-infected cell agglutination variant antigen (SICAvar), chromosome 9, centrally located; PKNH_0118500, type II SICAvar, chromosome 1, subtelomeric) are shown for comparison. Double peaking was seen in some samples; only the peak with highest amplitude was read for this analysis.

As further confirmation of our results, 7 *P. knowlesi* isolates detected by SICAvar were PCR amplified, and the products directly sequenced. The sequences exhibited high variability, as expected for variant antigens, even in this most conserved exon (Figure 3). Interestingly, 2 of the 7 sequences obtained harbored an insert encoding an additional 7 amino acids. These 2 forms were used to probe the current *P. knowlesi* reference genome (http://www.sanger.ac.uk/resources/downloads/protozoa/plasmodium-knowlesi.html). and both queries identified a number of distinct sequences in the reference genome (Supplementary Table 1).

**Figure 4.**
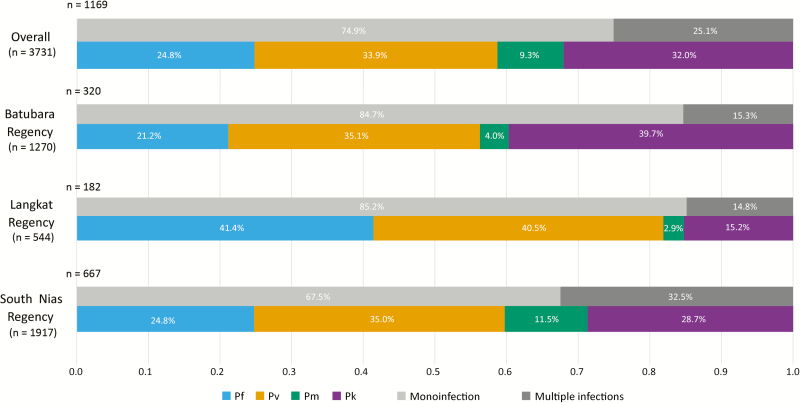
Proportion of *Plasmodium* species and multiplicity of infections by regency. The denominator for each site (total number of individuals tested) is given under the regency name, and the number of parasite-positive individuals is shown at the top-left of each bar graph. The horizontal axis represents the proportion of the total number of infections in each bar. Colored bars denote species; gray bars denote proportions of mixed-species infections identified in each site. Abbreviations: Pf , *Plasmodium falciparum*; Pk, *Plasmodium knowlesi*; Pm, *Plasmodium malariae*; Pv, *Plasmodium vivax*.

After this successful validation of the SICAvar PCR assay, we were able to deduce robust estimates of the contribution of each species to malaria infections in each of our 3 sites. The most abundant in both Langkat and South Nias were *P. falciparum* and/or *P. vivax*, as expected. However in Batubara, *P. knowlesi* (39.7%) was more abundant than *P. vivax* (35.1%) among our tested participants (Figure 4). Of patients reporting fever symptoms in the previous 72 hours, 22 were carrying *P. knowlesi* monoinfection by PCR, with another 47 having *P. knowlesi* double infections with *P. vivax* (n = 20), *P. falciparum* (n = 19), or *P. malariae* (n = 2) or various combinations of triple-species infection.

### Parasite Carriage

A total of 3731 individuals from Batubara (n = 1270), Langkat (n = 544), and South Nias (n = 1917) were included in the malaria screening (Supplementary Table 2). In these 3 regencies, 117 (9.2%), 98 (18.0%), and 397 (21.3%) individuals, respectively, were positive for *Plasmodium* infections by microscopy. Three species (*P. falciparum*, *P. vivax,* and *P. malariae*) were identified. A considerable number of participants with malaria-positive slides were negative by PCR subsequently performed on stored blood spots. Thus, the total number of patent infections confirmed by both microscopy and PCR decreased to 93 (8.1%), 74 (13.6%) and 169 (9.1%), respectively, indicating poor specificity of microscopy in South Nias in particular. Conversely, a substantial number of submicroscopic infections were identified by PCR alone, with a PCR-confirmed parasite carriage in 25.2%, 33.5%, and 34.8% of tested individuals, respectively, in the 3 sites (Table 2). All *Plasmodium* species with the exception of *P. ovale* spp. were detected among our samples by a combination of the rRNA gene and SICAvar PCR assays.

**Table 2.** Submicroscopic Infections in 1169 Participants With Positive PCR Results for *Plasmodium* spp.

Infection Type	Participants, No. (%)^a^	
	All PCR Positive	PCR Positive and Microscopy Negative
All *Plasmodium* infections	1169 (31.33)	833 (71.26)
*Plasmodium falciparum*	247 (6.62)	165 (14.11)
*Plasmodium vivax*	335 (8.97)	227 (19.41)
*Plasmodium malariae*	40 (1.07)	35 (2.99)
*Plasmodium knowlesi*	254 (6.80)	220 (18.81)
Mixed infections	293 (7.85)	186 (15.91)
No. of species by PCR		
1	876 (74.94)	647 (77.67)
2	256 (21.90)	163 (19.57)
3	35 (2.99)	21 (2.52)
4	2 (0.17)	2 (0.24)
PCR positive by regency		
Batubara	320/1270 (25.19)	227/320 (70.93)
Langkat	182/544 (33.45)	108/182 (59.34)
South Nias	667/1917 (34.79)	498/667 (74.66)

Abbreviation: PCR, polymerase chain reaction.

^a^Frequencies are shown together with relative frequency expressed as a percentage of all participants (N = 3731), all PCR-positive participants (n = 1169), or all participants with submicroscopic infections (n = 833). Top-row percentages read horizontally; percentages for other indented categories read vertically within the appropriate subgroup, apart from the regency-specific data (with denominators as shown).

### Carriage of Submicroscopic Infections

PCR analysis revealed that the majority of the 1169 infected individuals (71.3%) harbored submicroscopic parasites (Table 2). Among these submicroscopic infections, 77.7% (647 of 833) were single-species infections predominated by *P. vivax* (n = 227) and *P. knowlesi* (n = 220). Submicroscopic infections of any species were more often found in older individuals, the mean age in this group being 23.0 years, (95% confidence interval [CI], 21.7–24.3 years). The mean age of individuals with patent infections was significantly lower, at 18.0 years (95% CI, 16.2–19.7 years; *P* < .001, 2-sided t-test) (Table 3). Submicroscopic carriage was observed more often in Batubara (odds ratio, 1.67; 95% CI, 1.14–2.45) or South Nias (2.02; 1.43–2.85) than in Langkat. However, individuals with multispecies infections were not significantly older than those infected with a single species (*P* = .66, 2-sided *t* test).

**Table 3.** Age and Sex of Participants With Positive PCR Results for *Plasmodium* spp.

Age and Sex by *Plasmodium* Species^a^	PCR Positive, No. (%)
Parasite carriage by age group, y	
*P. falciparum*	
<5	24 (9.72)
5–14	91 (36.84)
>15	132 (53.44)
*P. vivax*	
<5	41 (12.24)
5–14	143 (42.69)
>15	151 (45.07)
*P. malariae*	
<5	4 (10.00)
5–14	18 (45.00)
>15	18 (45.00)
*P. knowlesi*	
<5	28 (11.02)
5–14	96 (37.80)
>15	130 (51.18)
Parasite carriage among female participants	608 (52.01)
*P. falciparum*	137 (55.47)
*P. vivax*	171 (51.04)
*P. malariae*	15 (37.50)
*P. knowlesi*	129 (50.79)

Abbreviation: PCR, polymerase chain reaction.

^a^Age and sex data are presented for single-species infection only.

## DISCUSSION

In this study, a total of 3731 febrile and nonfebrile residents of 3 regencies in North Sumatera province were screened for malaria infection by both microscopy and PCR detection of parasite DNA. Microscopy identified 612 infected participants, whereas PCR identified 1169 individuals harboring ≥1 of the 4 *Plasmodium* species identified: *P. falciparum*, *P. knowlesi*, *P. malariae,* and *P. vivax*. Using a novel assay developed for this study, which detects a conserved motif in the multicopy *sicavar* gene family, we found that *P. knowlesi* was present in 377 individuals (10.1%), including both patent and subpatent infections. *P. vivax* and *P. falciparum* were both frequently detected, occurring in 11.3% and 8.2% of all individuals tested.

Although *P. knowlesi* infection has been widely recorded in Southeast Asia, only a handful of confirmed cases have been from Indonesia, from Kalimantan, eastern Borneo [8, 24], and, more recently, 20 cases from Aceh province, on the northwest extremity of Sumatera [5]. This latter study of 1532 individuals used a combination of passive and reactive case detection to identify a total of 20 *P. knowlesi*, 15 *P. vivax,* and 8 *P. falciparum* infections, almost all of which were symptomatic. This contrasts with our findings of more frequent parasite carriage, and a significant proportion of subpatent and asymptomatic infections.

Malaria transmission intensity is much lower in Aceh than in North Sumatera, and this neighboring province is closer to eliminating the disease. North Sumatera’s natural forests have been affected by deforestation in the last few years. Residents of rural districts may live in close proximity to semiforested, forested, or plantation areas, with a high likelihood of forest exposure, but may not have adequate access to health facilities and antimalarial medication. In the Aceh study, the more frequent occurrence of symptoms in persons with knowlesi malaria reflects observations in Malaysian Borneo where, as in Aceh, other *Plasmodium* species have decreased in prevalence over the past decade [4, 25]. In North Sumatera, as our data show, *P. falciparum* and *P. vivax* remain common and it may be that acquired immunity to these human parasites protects individuals subsequently infected with *P. knowlesi*, although asymptomatic infections have also been reported in Sabah [7].

Our novel PCR assay identified an unexpectedly large number of *P. knowlesi* infections, and so we made some effort to validate its sensitivity and specificity (Figure 2). SICAvar genes encode an antigen family unique to *P. knowlesi*, estimated to number >100 members, including both multiexon and truncated forms randomly distributed across all 14 chromosomes [23]. SICAvar proteins undergo antigenic variation in the course of a single infection [26, 27] and are likely to play a key role in maintaining chronic parasitemia in semi-immune hosts. Sequencing of a handful of *sicavar* amplicons from our samples confirmed nucleotide diversity in our short target sequence, double peaking indicative of multiple loci being amplified in some cases, and distinguished a variant form with a 7–amino acid insert (Figure 3). Probing the *P. knowlesi* reference genome with these 2 forms generated many hits with both length variants, including both types 1 and 2 loci (Supplementary Table 1). These findings suggest our assay is performing as hoped and is a useful tool for identifying *P. knowlesi* infections among complex mixtures of *Plasmodium* species.

The national malaria control program focuses on case management through passive surveillance at primary health centers, deploying microscopy or RDTs to detect malaria cases to be treated [9]. In our study, microscopy identified many *Plasmodium* spp. infections, but lacked accuracy in distinguishing among the 4 species present, as previously reported in Malaysia [28]. Although personnel differed between sites, identification of the less common species was problematic in all sites. *P. malariae* was only detected in 1 case and *P. knowlesi* was left unrecognized. In South Nias, microscopy results showed poor specificity (Supplementary Table 2), leading to a number of false-positives, whereas specificity was good at the other 2 sites. RDTs have limitations for detection of *P. knowlesi* because they include only *P. falciparum* and *P. vivax* parasite lactate dehydrogenase monoclonal antibodies [11]. Most importantly, almost half of the infections detected with PCR were not identified by either of the conventional tests. Therefore, although RDTs and microscopy remain satisfactory for diagnosis of symptomatic falciparum and vivax malaria requiring treatment, these are not adequate tools for malaria elimination and control activities, because submicroscopic *Plasmodium* carriage is associated with subsequent transmission to mosquitoes [13].

On the other hand, molecular tests are highly sensitive and specific, provide the capacity to detect low-density infections missed by microscopy or RDTs, and are well established for detection of human malaria infections [11, 29]. To overcome cross-amplification of *P. vivax* isolates with ribosomal gene PCR assays [3, 19], Ghinai et al [30] have recently described a PCR-sequencing approach to detect *P. knowlesi cytb* DNA, which also provided satisfactory sensitivity. However, our *sicavar* target assay provides greater sensitivity for *P. knowlesi* identification in our hands and was effective in detecting submicroscopic parasites. This is a species-specific test, because the *sicavar* gene family is unique to *P. knowlesi* (Figure 2). Because of this improved specificity and sensitivity, we can confidently report moderate numbers of *P. knowlesi* coinfections with *P. vivax* in the present study.

Despite the aim to achieve malaria elimination by 2020 in Sumatera, our study demonstrated that a substantial number of individuals in our study areas carried parasites. *P. falciparum* contributed one-fifth of infections, and *P. vivax* was seen slightly more often. Interestingly, our findings also demonstrate that *P. knowlesi* carriage is not uncommon. Many *P. knowlesi*–infected individuals harbored additional *Plasmodium* species (Table 1), in contrast to areas in Malaysian Borneo where *P. vivax* and *P. falciparum* are now very scarce [4, 7]. Multiple-species infections in our study were equally distributed across all age groups with both female and male subjects exposed to a similar risk of infection (data not shown), whereas submicroscopic infections were more common in older individuals (Table 2), suggesting a role for acquired immunity [31]. The observation of asymptomatic *P. knowlesi* infections in our study is consistent with recent findings in Malaysia [7] but does not necessarily support the occurrence of human-mosquito transmission of *P. knowlesi.*

Macaques were present in all sites, and the communities shared established risk factors for malaria transmission by forest-dwelling *Anopheles,* thought to be the vector of *P. knowlesi*. These data may suggest that acquired immunity permits sustainable chronic infections with this simian parasite. Cross-protection among the 4 human *Plasmodium* species may maintain overall parasite density at low levels [31], which could also plausibly apply to *P. knowlesi,* particularly in settings where the closely related *P. vivax* is present. One weakness of our study is the lack of a systematic sampling procedure, which may have introduced bias; as we had not previously worked in these 3 regions, methods were adapted for each site to reflect the facilities available and particular local challenges. For South Nias, these included frequent sea journeys in small boats. Future studies could deploy a more systematic approach and collect sufficient data to better explain and characterize asymptomatic infections, now that community contacts have been established and baseline information is available.

Our study demonstrated the importance of submicroscopic infections of 4 *Plasmodium* species, including *P. knowlesi*, to malaria transmission in North Sumatera. There is an urgent need for the national malaria program to include in malaria guidelines the recommendation that microscopists are trained to identify *P. knowlesi* infection in Indonesian clinics. Molecular detection of infection is also needed, to strengthen control and elimination programs by accurately defining the true extent of the malaria reservoir, so as to achieve the current goal of elimination [32].

## Supplementary Data

Supplementary materials are available at *The Journal of Infectious Diseases* online. Consisting of data provided by the authors to benefit the reader, the posted materials are not copyedited and are the sole responsibility of the authors, so questions or comments should be addressed to the corresponding author.

## Supplementary Material

Supplementary Tables

## Notes

***Acknowledgments.*** We thank the participants, their families, and the community health workers and village leaders who gave their cooperation and support to the work, and we thank Gisela Henriques for her contribution to the field studies in South Nias. We are grateful to David Conway, Clemens Kocken, and Balbir Singh for providing control DNA from primate malaria species and to Franziska Mohring, who provided cultured *P. knowlesi* parasites. The Public Health England Malaria Reference Laboratory, London, kindly provided validated DNA controls from human malaria species. We thank Kevin Baird, Rob Moon, and Janet Cox-Singh for helpful discussions. Two anonymous reviewers provided excellent suggestions for improving the manuscript.

***Financial support.*** This work was supported by the University of Sumatera Utara, Medan, and the London School of Hygiene & Tropical Medicine.

***Potential conflicts of interest.*** All authors: No reported conflicts. All authors have submitted the ICMJE Form for Disclosure of Potential Conflicts of Interest. Conflicts that the editors consider relevant to the content of the manuscript have been disclosed.
