# Supplementary material for: Contribution of Plasmodium knowlesi to Multispecies Human Malaria Infections in North Sumatera, Indonesia
Source: J Infect Dis. 2017 Feb 13;215(7):1148–55. doi: 10.1093/infdis/jix091 (PMC5426374; doi:10.1093/infdis/jix091)
Supplement: Supplementary Tables [file jix091_suppl_Supplementary_Tables.docx]

**Supplementary Table 1.** *Low coding diversity among* SICAvar *loci around the target sequence used for amplification to detect* P. knowlesi *in study participants.*

| Query | Locus | Score | E | Predicted amino acid sequence |
| --- | --- | --- | --- | --- |
| 1 | [PKNH_0407200](http://plasmodb.org/plasmo/showRecord.do?name=GeneRecordClasses.GeneRecordClass&project_id=PlasmoDB&source_id=PKNH_0407200) | [86.7](http://plasmodb.org/plasmo/showApplication.do#PKNH_0407200) | 9e-22 | SVQEQVLDHVQQDGPHEYQLVKERKPRSAPTRTKRSGPVN |
| 1 | [PKNH_1306200](http://plasmodb.org/plasmo/showRecord.do?name=GeneRecordClasses.GeneRecordClass&project_id=PlasmoDB&source_id=PKNH_1306200) | [80.9](http://plasmodb.org/plasmo/showApplication.do#PKNH_1306200) | 7e-20 | SVQEQVLDHVQQDSSHEYQLVKERKPRSAPTRTKRSGPVN |
| 1 | [PKNH_1454800](http://plasmodb.org/plasmo/showRecord.do?name=GeneRecordClasses.GeneRecordClass&project_id=PlasmoDB&source_id=PKNH_1454800) | [80.9](http://plasmodb.org/plasmo/showApplication.do#PKNH_1454800) | 8e-20 | SVQEQVLDHVQQDSPHEYQLVKERKPRSAPTRTKRSGRVN |
| 1 | [PKNH_1326600](http://plasmodb.org/plasmo/showRecord.do?name=GeneRecordClasses.GeneRecordClass&project_id=PlasmoDB&source_id=PKNH_1326600) | [80.1](http://plasmodb.org/plasmo/showApplication.do#PKNH_1326600) | 2e-19 | SVQEQLLDHVQQDSSHEYQLVKERKPRSAPTRTKRSGPVN |
| 1 | [PKNH_0825500](http://plasmodb.org/plasmo/showRecord.do?name=GeneRecordClasses.GeneRecordClass&project_id=PlasmoDB&source_id=PKNH_0825500) | [79.3](http://plasmodb.org/plasmo/showApplication.do#PKNH_0825500) | 3e-19 | SVQEQVLDHVEEAGPHEYRLVKERKPRSAPTRTKRSGPVN |
| 1 | [PKNH_0837900](http://plasmodb.org/plasmo/showRecord.do?name=GeneRecordClasses.GeneRecordClass&project_id=PlasmoDB&source_id=PKNH_0837900) | [79.3](http://plasmodb.org/plasmo/showApplication.do#PKNH_0837900) | 3e-19 | SVQEQVLDHVQQDSSHEYRLVKERKPRSAPTRTKRSGPVN |
| 1 | [PKNH_0940500](http://plasmodb.org/plasmo/showRecord.do?name=GeneRecordClasses.GeneRecordClass&project_id=PlasmoDB&source_id=PKNH_0940500) | [79.3](http://plasmodb.org/plasmo/showApplication.do#PKNH_0940500) | 3e-19 | SVQEQVLDHVQQDSSHEYRLVKERKPRSAPTRTKRSGPVN |
| 1 | [PKNH_1120600](http://plasmodb.org/plasmo/showRecord.do?name=GeneRecordClasses.GeneRecordClass&project_id=PlasmoDB&source_id=PKNH_1120600) | [79.3](http://plasmodb.org/plasmo/showApplication.do#PKNH_1120600) | 3e-19 | SVQEQVLDHLQQDSSHEYQLVKERKPRSAPTRTKRSGPVN |
| 1 | [PKNH_1459300](http://plasmodb.org/plasmo/showRecord.do?name=GeneRecordClasses.GeneRecordClass&project_id=PlasmoDB&source_id=PKNH_1459300) | [79.3](http://plasmodb.org/plasmo/showApplication.do#PKNH_1459300) | 3e-19 | SVQEQVLDHVEEAGPHEYRLVKERKPRSAPTRTKRSGPVN |
| 1 | [PKNH_0837800](http://plasmodb.org/plasmo/showRecord.do?name=GeneRecordClasses.GeneRecordClass&project_id=PlasmoDB&source_id=PKNH_0837800) | [79.0](http://plasmodb.org/plasmo/showApplication.do#PKNH_0837800) | 4e-19 | SVQEQLLDHVQQDSSHEYQLVKERKPRSAPTRTKRSGPVN |
| 1 | [PKNH_0907700](http://plasmodb.org/plasmo/showRecord.do?name=GeneRecordClasses.GeneRecordClass&project_id=PlasmoDB&source_id=PKNH_0907700) | [79.0](http://plasmodb.org/plasmo/showApplication.do#PKNH_0907700) | 4e-19 | LVQEQVLDHVQQDSSHEYQLVKERKPRSAPTRTKRSGPVN |
| 1 | [PKNH_1138600](http://plasmodb.org/plasmo/showRecord.do?name=GeneRecordClasses.GeneRecordClass&project_id=PlasmoDB&source_id=PKNH_1138600) | [79.0](http://plasmodb.org/plasmo/showApplication.do#PKNH_1138600) | 4e-19 | SVQEQVLDHVEEAGPHEYQLVKERKPRSTPTRTKRSGPVN |
| 1 | [PKNH_1463800](http://plasmodb.org/plasmo/showRecord.do?name=GeneRecordClasses.GeneRecordClass&project_id=PlasmoDB&source_id=PKNH_1463800) | [79.0](http://plasmodb.org/plasmo/showApplication.do#PKNH_1463800) | 3e-19 | SVQEQVLDHVEEAGPHEYRLVKERKPRSAPTRTKRSGPVN |
| 1 | [PKNH_0418900](http://plasmodb.org/plasmo/showRecord.do?name=GeneRecordClasses.GeneRecordClass&project_id=PlasmoDB&source_id=PKNH_0418900) | [78.6](http://plasmodb.org/plasmo/showApplication.do#PKNH_0418900) | 6e-19 | SVQEQVLDHVQQDSSHEYKLVKERKPRSAPTRTKRSGPVN |
| 1 | [PKNH_0423700](http://plasmodb.org/plasmo/showRecord.do?name=GeneRecordClasses.GeneRecordClass&project_id=PlasmoDB&source_id=PKNH_0423700) | [78.2](http://plasmodb.org/plasmo/showApplication.do#PKNH_0423700) | 8e-19 | SVQEQVLDHVQQDSSHEYRLVKERKPRSAPTRTKRSGPVN |
| 1 | [PKNH_0500100](http://plasmodb.org/plasmo/showRecord.do?name=GeneRecordClasses.GeneRecordClass&project_id=PlasmoDB&source_id=PKNH_0500100) | [78.2](http://plasmodb.org/plasmo/showApplication.do#PKNH_0500100) | 1e-19 | SVQEQVLDHVEEAGPHEYRLVKERKPRSAPTRTKRSGPVN |
| 1 | [PKNH_0808000](http://plasmodb.org/plasmo/showRecord.do?name=GeneRecordClasses.GeneRecordClass&project_id=PlasmoDB&source_id=PKNH_0808000) | [78.2](http://plasmodb.org/plasmo/showApplication.do#PKNH_0808000) | 6e-19 | SVQEQVLDHVEEAGPHEYRLVKERKPRSAPTRTKRSGPVN |
| 1 | [PKNH_0818100](http://plasmodb.org/plasmo/showRecord.do?name=GeneRecordClasses.GeneRecordClass&project_id=PlasmoDB&source_id=PKNH_0818100) | [78.2](http://plasmodb.org/plasmo/showApplication.do#PKNH_0818100) | 7e-19 | SVQEQVLDHVQQDSSHEYRLVKERKPRSAPTRTKRSGPVN |
| 1 | [PKNH_0830000](http://plasmodb.org/plasmo/showRecord.do?name=GeneRecordClasses.GeneRecordClass&project_id=PlasmoDB&source_id=PKNH_0830000) | [78.2](http://plasmodb.org/plasmo/showApplication.do#PKNH_0830000) | 3e-19 | SVQEQVLDHVQQDSSHEYRLVKERKPRSAPTRTKRSGPVN |
| 1 | [PKNH_1272200](http://plasmodb.org/plasmo/showRecord.do?name=GeneRecordClasses.GeneRecordClass&project_id=PlasmoDB&source_id=PKNH_1272200) | [78.2](http://plasmodb.org/plasmo/showApplication.do#PKNH_1272200) | 7e-19 | SVQEQVLDHVQQDSSHEYRLVKERKPRSAPTRTKRSGPVN |
|  |  |  |  |  |
| 2 | [PKNH_0932000](http://plasmodb.org/plasmo/showRecord.do?name=GeneRecordClasses.GeneRecordClass&project_id=PlasmoDB&source_id=PKNH_0932000) | [99.8](http://plasmodb.org/plasmo/showApplication.do#PKNH_0932000) | 3e-25 | SVQEQLLDHVDEGASHEYQLVKERKPRSAPTRTKRSGRDPAGGGRVN |
| 2 | [PKNH_1427700](http://plasmodb.org/plasmo/showRecord.do?name=GeneRecordClasses.GeneRecordClass&project_id=PlasmoDB&source_id=PKNH_1427700) | [95.9](http://plasmodb.org/plasmo/showApplication.do#PKNH_1427700) | 7e-24 | SVQEQLLDHADEGASHEYRLVKERKPRSAPTRTKRSGRDPAGGGRVN |
| 2 | [PKNH_1149800](http://plasmodb.org/plasmo/showRecord.do?name=GeneRecordClasses.GeneRecordClass&project_id=PlasmoDB&source_id=PKNH_1149800) | [92.4](http://plasmodb.org/plasmo/showApplication.do#PKNH_1149800) | 8e-24 | SVQEQLLDHVEEAGPHEYQLVKERKPRSAPTRTKRSGRDPAGGGRVN |
| 2 | [PKNH_0900100](http://plasmodb.org/plasmo/showRecord.do?name=GeneRecordClasses.GeneRecordClass&project_id=PlasmoDB&source_id=PKNH_0900100) | [88.6](http://plasmodb.org/plasmo/showApplication.do#PKNH_0900100) | 2e-21 | SVQEQVLGHVDEGAAHEYRLVKERKPRSAPTRTKRSGRDPGGSGRVN |
| 2 | [PKNH_0929800](http://plasmodb.org/plasmo/showRecord.do?name=GeneRecordClasses.GeneRecordClass&project_id=PlasmoDB&source_id=PKNH_0929800) | [88.2](http://plasmodb.org/plasmo/showApplication.do#PKNH_0929800) | 4e-21 | SVQEHLLDHVEEAGPHEYRLVKERKPRSAPTRTKRSGRDPAGGGRVN |
| 2 | [PKNH_0311900](http://plasmodb.org/plasmo/showRecord.do?name=GeneRecordClasses.GeneRecordClass&project_id=PlasmoDB&source_id=PKNH_0311900) | [87.4](http://plasmodb.org/plasmo/showApplication.do#PKNH_0311900) | 2e-21 | SVQEQLLDHVEEAGPHEYRLVKERKPRSAPTRTKRSGRDPAGSGRVN |
| 2 | [PKNH_0818000](http://plasmodb.org/plasmo/showRecord.do?name=GeneRecordClasses.GeneRecordClass&project_id=PlasmoDB&source_id=PKNH_0818000) | [86.3](http://plasmodb.org/plasmo/showApplication.do#PKNH_0818000) | 4e-21 | SVQEQVLDHVQQDSSHEYRLVKERKPRSAPTRTKRSGRDPAGSGRVN |
| 2 | [PKNH_0216500](http://plasmodb.org/plasmo/showRecord.do?name=GeneRecordClasses.GeneRecordClass&project_id=PlasmoDB&source_id=PKNH_0216500) | [84.7](http://plasmodb.org/plasmo/showApplication.do#PKNH_0216500) | 7e-22 | SVQEQVLDHVQQDSSHEYRLVKERKPRSAPTRTKRSGRDPAGSGRVN |
| 2 | [PKNH_0400200](http://plasmodb.org/plasmo/showRecord.do?name=GeneRecordClasses.GeneRecordClass&project_id=PlasmoDB&source_id=PKNH_0400200) | [84.7](http://plasmodb.org/plasmo/showApplication.do#PKNH_0400200) | 4e-21 | SVQEQVLDHVEEAGSHEYQLVKERKPPSVPARTKRSARDPAGGGRVN |
| 2 | [PKNH_0735100](http://plasmodb.org/plasmo/showRecord.do?name=GeneRecordClasses.GeneRecordClass&project_id=PlasmoDB&source_id=PKNH_0735100) | [84.3](http://plasmodb.org/plasmo/showApplication.do#PKNH_0735100) | 6e-21 | SVQEQLLDHVEEAGPHEYRLVKERKPPSVPARTKRSGRDPAGGGRVN |
| 2 | [PKNH_1247100](http://plasmodb.org/plasmo/showRecord.do?name=GeneRecordClasses.GeneRecordClass&project_id=PlasmoDB&source_id=PKNH_1247100) | [84.3](http://plasmodb.org/plasmo/showApplication.do#PKNH_1247100) | 5e-21 | SVQEQLLDHVEEAGPHEYQLVKERKPPSVPARTKRSARDPAGGGRVN |
| 2 | [PKNH_0100300](http://plasmodb.org/plasmo/showRecord.do?name=GeneRecordClasses.GeneRecordClass&project_id=PlasmoDB&source_id=PKNH_0100300) | [84.0](http://plasmodb.org/plasmo/showApplication.do#PKNH_0100300) | 8e-21 | SVQEQLLDHVEEAGPHEYQLVKERKPPSVPARTKRSGRDPADGGRVN |
| 2 | [PKNH_0800200](http://plasmodb.org/plasmo/showRecord.do?name=GeneRecordClasses.GeneRecordClass&project_id=PlasmoDB&source_id=PKNH_0800200) | [83.6](http://plasmodb.org/plasmo/showApplication.do#PKNH_0800200) | 1e-20 | SVQEQLLDHVQQDSSHEYQLVKERKPPSVPARTKRSARDPAGGGRVN |
| 2 | [PKNH_1200200](http://plasmodb.org/plasmo/showRecord.do?name=GeneRecordClasses.GeneRecordClass&project_id=PlasmoDB&source_id=PKNH_1200200) | [82.4](http://plasmodb.org/plasmo/showApplication.do#PKNH_1200200) | 3e-20 | SVQEQLLDHVEEAGPHEYRLVKERKPPSVPARTKRSGLDPAGGGRVN |
| 2 | [PKNH_0623800](http://plasmodb.org/plasmo/showRecord.do?name=GeneRecordClasses.GeneRecordClass&project_id=PlasmoDB&source_id=PKNH_0623800) | [82.0](http://plasmodb.org/plasmo/showApplication.do#PKNH_0623800) | 4e-20 | SVQEQVLDHVDEAGPHEYRLVKERKPPSVPARTKRSGLDPAGGGRVN |
| 2 | [PKNH_1357200](http://plasmodb.org/plasmo/showRecord.do?name=GeneRecordClasses.GeneRecordClass&project_id=PlasmoDB&source_id=PKNH_1357200) | [81.3](http://plasmodb.org/plasmo/showApplication.do#PKNH_1357200) | 9e-20 | SVQEQLLDHVEEAGPHEYRLVKERKPPSVPARTKRSGRDPAGDGRVN |
| 2 | [PKNH_0825600](http://plasmodb.org/plasmo/showRecord.do?name=GeneRecordClasses.GeneRecordClass&project_id=PlasmoDB&source_id=PKNH_0825600) | [80.1](http://plasmodb.org/plasmo/showApplication.do#PKNH_0825600) | 7e-19 | SVQEQVLDHVQQDSSHEYRLFKERKPRYVPTRTKRSGRDPAGSGRVN |
| 2 | [PKNH_1443500](http://plasmodb.org/plasmo/showRecord.do?name=GeneRecordClasses.GeneRecordClass&project_id=PlasmoDB&source_id=PKNH_1443500) | [80.1](http://plasmodb.org/plasmo/showApplication.do#PKNH_1443500) | 8e-19 | SVQEQLLDHVEEAGSHEYRLFKERKRRSAPTRTKRSGRDPARSGRVN |
| 2 | [PKNH_1112700](http://plasmodb.org/plasmo/showRecord.do?name=GeneRecordClasses.GeneRecordClass&project_id=PlasmoDB&source_id=PKNH_1112700) | [75.1](http://plasmodb.org/plasmo/showApplication.do#PKNH_1112700) | 1e-16 | SVQEQVLDHVDEGASHEYRLVKERKPRSAPTRTKRSG-------HVN |

Two query sequences from SICAvar sequencing results shown in Figure 3 were used to interrogate the *P. knowlesi* reference genome using BLAST. The top twenty hits from each query are shown; there is no redundancy between the two queries.

Shaded sequences denote Type II SICAvar loci.

Query 1: SVQEQVLDHVQQDGPHEYQLVKERKPRSAPTRTKRSGPVN Length=40

Query 2: SVQEQLLDHVDEGASHEYQLVKERKPRSAPTRTKRSGRDPAGGGRVN Length=47

Score: alignment scores from BLAST (bits)

E: probability of achieving observed level of similarity by chance

Searches performed at: <http://plasmodb.org/plasmo/showQuestion.do?questionFullName=UniversalQuestions.UnifiedBlast>

**Supplementary Table 2. Detection of *Plasmodium* species by microscopy and nested PCR***

|  | **Microscopy** | **Detection by Nested PCR** | | | | | | | | | | | | | | | | |
| --- | --- | --- | --- | --- | --- | --- | --- | --- | --- | --- | --- | --- | --- | --- | --- | --- | --- | --- |
|  |  | **Pf** | **Pv** | **Pm** | **Pk** | **Pf**  **Pv** | **Pf Pm** | **Pf**  **Pk** | **Pf**  **Pv Pm** | **Pf**  **Pv Pk** | **Pf Pm Pk** | **Pf**  **Pv**  **Pm**  **Pk** | **Pv Pm** | **Pv Pk** | **Pv Pm Pk** | **Pm Pk** | **Neg** | ***Total*** |
| **Batubara Regency** | **Pf** | 18 | - | - | - | 3 | - | 5 | - | - | - | - | - | - | - | - | 8 | *34* |
|  | **Pv** | 1 | 37 | - | 4 | 3 | - | - | - | 3 | - | - | - | 11 | - | - | 15 | *74* |
|  | **Pf Pv** | 1 | 3 | - | - | 2 | - | - | - | - | - | - | - | 1 | 1 | - | 1 | *9* |
|  | **Pm** | - | - | - | - | - | - | - | - | - | - | - | - | - | - | - | - | *-* |
|  | **Neg** | 34 | 53 | 10 | 110 | 4 | - | 5 | - | - | - | - | 3 | 7 | - | 1 | 926 | *1153* |
|  | ***Total*** | *54* | *93* | *10* | *114* | *12* | *-* | *10* | *-* | *3* | *-* | *-* | *3* | *19* | *1* | *1* | *950* | ***127*** |
| **Langkat Regency** | **Pf** | 18 | 3 | - | 3 | 2 | - | - | - | - | - | - | - | - | - | - | 15 | *41* |
|  | **Pv** | 3 | 24 | - | 1 | 5 | - | - | - | - | - | - | - | 5 | - | - | 7 | *45* |
|  | **Pf Pv** | 4 | 1 | - | - | 1 | - | - | - | - | - | - | - | 3 | - | - | 2 | *11* |
|  | **Pm** | - | - | 1 | - | - | - | - | - | - | - | - | - | - | - | - | - | *1* |
|  | **Neg** | 45 | 34 | 2 | 16 | 5 | 2 | 1 | - | 1 | - | - | - | 1 | - | 1 | 338 | *446* |
|  | ***Total*** | *70* | *62* | *3* | *20* | *13* | *2* | *1* | *-* | *1* | *-* | *-* | *-* | *9* | *-* | *1* | *362* | ***544*** |
| **South Nias Regency** | **Pf** | 26 | 22 | 3 | 18 | 7 | 2 | 14 | 1 | 1 | 2 | - | 2 | 12 | 2 | 2 | 154 | *268* |
|  | **Pv** | 8 | 16 | - | 7 | 2 | 1 | 1 | - | 2 | - | - | 1 | 1 | 1 | 1 | 64 | *105* |
|  | **Pf Pv** | 3 | 2 | 1 | 1 | 2 | - | - | - | - | 1 | - | 2 | 2 | - | - | 12 | *26* |
|  | **Pm** | - | - | - | - | - | - | - | - | - | - | - | - | - | - | - | - | *-* |
|  | **Neg** | 86 | 140 | 23 | 94 | 25 | 4 | 26 | - | 8 | 3 | 2 | 25 | 34 | 9 | 19 | 1020 | *1518* |
|  | ***Total*** | *123* | *180* | *27* | *120* | *36* | *7* | *41* | *1* | *11* | *6* | *2* | *30* | *49* | *12* | *22* | *1250* | ***1917*** |

Pf=*P. falciparum*, Pv=*P.vivax*, Pm=*P.malariae*, Pk=*P.knowlesi*

*Nested PCR targeting the 18 ssu rRNA gene (plus SICAvar gene for *P.knowlesi* infection)
